# 204. *Clostridioides difficile* screening among asymptomatic carriers admitted for bone marrow transplantation: Navigating optimal stewardship strategies

**DOI:** 10.1093/ofid/ofae631.062

**Published:** 2025-01-29

**Authors:** Justin Hofmann, Alma Habib, Nikki Tran, Sarah MacDowell, Hannah Choe, Christina Liscynesky, Zeinab El Boghdadly

**Affiliations:** Ohio State University Medical Center/Nationwide Children's Hospital, Columbus, OH; The Ohio State University, Columbus, Ohio; The Ohio State Wexner Medical Center, Columbus, Ohio; The Ohio State University, Columbus, Ohio; Ohio State University Wexner Medical Center, Columbus, Ohio; The Ohio State Univiersity Wexner Medical Center, Columbus, Ohio; The Ohio State University, Columbus, Ohio

## Abstract

**Background:**

Toxigenic Clostridioides difficile (CD) approximately colonizes 11-39% of allogeneic and 13% of autologous hematopoietic stem cell transplantation (HCT) candidates. Colonization increases risk for CD infection (CDI). Thus, in 2018, our institution established universal CD PCR screening on admission to the HCT unit to decrease hospital onset CDI rates. CD colonized patients received primary prophylaxis with twice daily oral vancomycin (OV) if antibiotics were initiated. Here, we share our experience with this process and subsequent diagnostic and therapeutic stewardship interventions.

**Figure:**
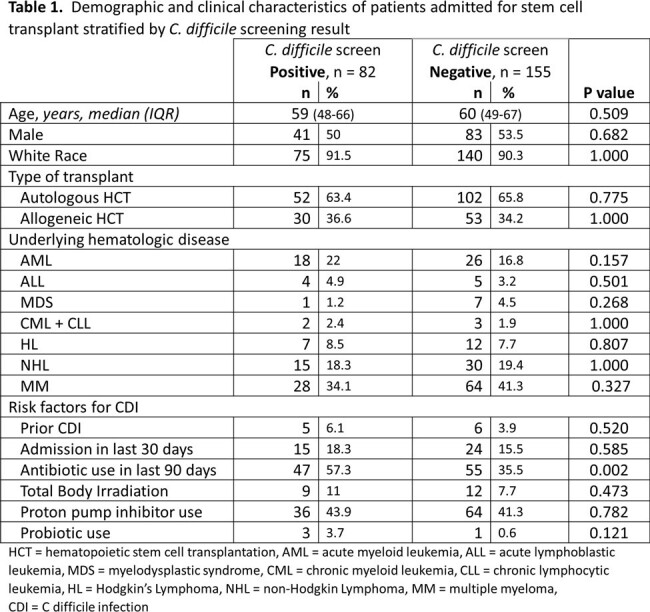

**Methods:**

We retrospectively analyzed adult HCT patients who had stool CD PCR screening within 72 hours of HCT admission between 12/2018 to 8/2021. Outcomes were stratified by CD screen positive vs negative within 100 days post-HCT. CD PCR was the methodology used for CDI diagnosis during this time frame.

**Figure:**
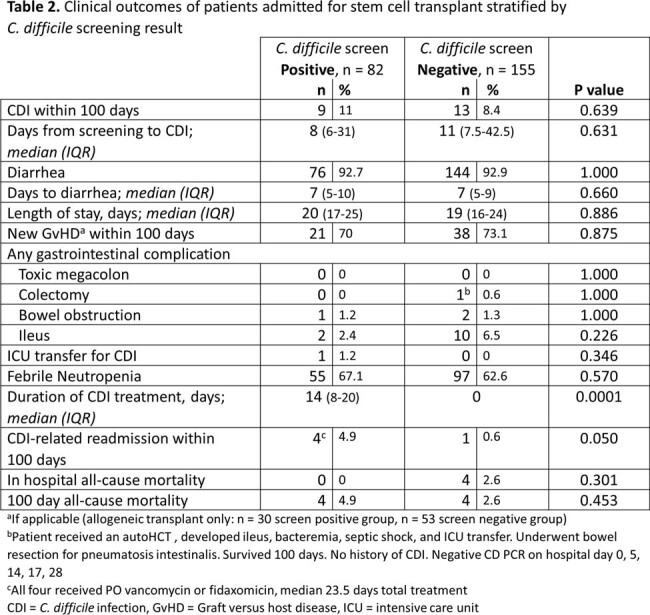

**Results:**

We reviewed 237 patients (autoHCT, n=154; alloHCT, n=83). Patient characteristics are described in Table 1. Thirty alloHCT and 52 autoHCT patients screened positive for CD. Prior antibiotic use was observed more in the CD colonized group. Nine (11%) screen-positive patients developed CDI in 100 days versus 13 (8.4%) screen-negative. No significant differences in other outcomes were noted (Table 2). Diarrhea occurred in 76 (92.7%) of the screen-positive patients and 144 (92.9%) of the screen-negative. A second CD PCR test was ordered in 18 patients from the positive group and 129 from the negative group; 16 of 18 (88.9%) converted to a negative result without any CD-directed treatment and 9 of 129 (7%) became positive. In 9/2023, CD screening policy ceased in lieu of a 2-step CD PCR plus toxin A/B enzyme immunoassay (EIA) for clinical suspicion of CDI. This change reduced CDI rate and antibiotic utilization (Figures 1,2).

**Figure 1.**
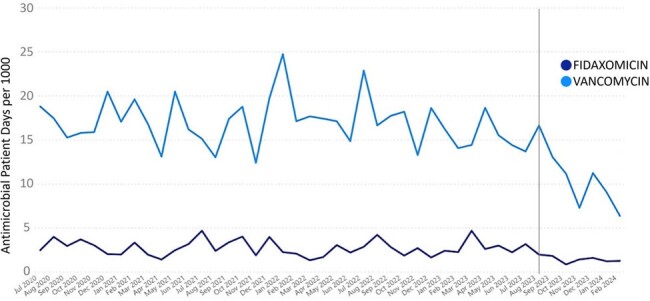
Decrease in anti-CDI antimicrobial utilization in the bone marrow transplant unit over time following two-step CD testing implementation in 9/2023

**Conclusion:**

Regardless of CD carrier status, diarrhea is an inevitable early post-HCT adverse effect due to commonly used conditioning chemotherapies for autoHCT and alloHCT. Screening for CD carriage on HCT admission may not accurately assess risk of future CDI and may only reflect transient carriage in some patients. Better CDI diagnostic tools such as 2-step test is needed in this population to avoid over diagnosis and treatment of CDI.

**Figure 2.**
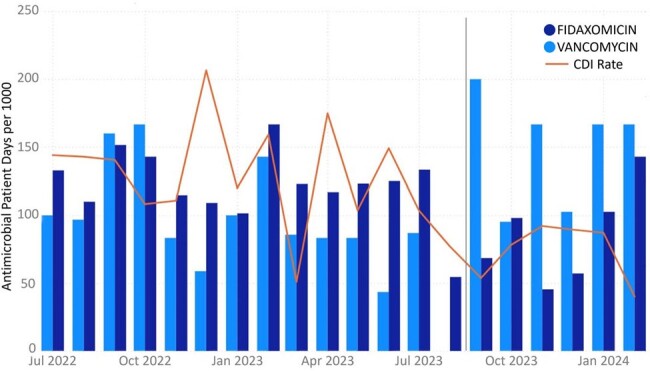
Decrease in anti-CDI antimicrobial utilization and CDI rate of all patients (including non-transplant patients) admitted to the cancer center over time following two-step CD testing implementation

**Disclosures:**

**Hannah Choe, MD**, Abbvie: Advisor/Consultant|Actinium: Advisor/Consultant|Incyte: Advisor/Consultant|Ironwood: Advisor/Consultant|Regimmune: Advisor/Consultant|Sanofi: Advisor/Consultant

